# The Value of Interleukin-6 among Several Inflammatory Markers as a Predictor of Respiratory Failure in COVID-19 Patients

**DOI:** 10.3390/diagnostics11081327

**Published:** 2021-07-23

**Authors:** Mitsuhiro Fujino, Michiyo Ishii, Takuya Taniguchi, Hiroya Chiba, Masaki Kimata, Masahito Hitosugi

**Affiliations:** 1 Department of Critical Care and Emergency Medicine, Otsu City Hospital, 2-9-9, Motomiya, Otsu 520-0804, Japan; chiba19840720@yahoo.co.jp; 2 Department of Internal Medicine, Otsu City Hospital, 2-9-9, Motomiya, Otsu 520-0804, Japan; michiishii@gmail.com; 3 Department of Cardiology, Otsu City Hospital, 2-9-9, Motomiya, Otsu 520-0804, Japan; takuya-t@koto.kpu-m.ac.jp (T.T.); mkimata@koto.kpu-m.ac.jp (M.K.); 4 Department of Legal Medicine, Shiga University of Medical Science, Tsukinowa, Seta, Otsu 520-2192, Japan; hitosugi@belle.shiga-med.ac.jp

**Keywords:** inflammatory marker, interleukin-6, novel coronavirus disease, respiratory failure

## Abstract

Patients with coronavirus disease 2019 (COVID-19) develop severe respiratory failure within a short period during the clinical course. It is essential to predict respiratory deterioration in the short term. We investigated the use of inflammatory markers to predict respiratory distress within three days from their analysis in COVID-19 patients. This retrospective observational study included 81 patients admitted with COVID-19. Patients were divided into two groups according to whether the maximum fraction of inspired oxygen (FiO_2_) for three days from the blood marker measurements was ≥0.4 (high FiO_2_ group; HFG) or <0.4 (low FiO_2_ group; LFG). Interleukin-6 (IL-6), C-reactive protein (CRP), lactate dehydrogenase (LDH), white blood cell, D-dimer, and creatinine levels were compared between the two groups. The levels of all markers were significantly higher in HFG patients. Areas under the receiver operating characteristic curve of IL-6, CRP, and LDH had high values of 0.85, 0.82, and 0.81, respectively. The odds ratio of IL-6 which was crude and adjusted for dexamethasone administration initiated before laboratory measurement, showed the high value of 29.1 (5.6–295.6) and 53.9 (4.5–3242.8), respectively. IL-6 can be used as a reliable marker for predicting respiratory illness within three days after assessment.

## 1. Introduction

Coronavirus disease 2019 (COVID-19) was first reported in Wuhan, China, in December 2019, and WHO declared it a pandemic in January 2020 [1]. This pandemic represents a global crisis for public health. The clinical manifestations of COVID-19 are wide-ranging, from asymptomatic to severe viral pneumonia such as acute respiratory distress syndrome (ARDS). Some COVID-19 patients experience respiratory deterioration over a short period of time during the clinical course. Thus, it is essential to identify patients who are likely to develop severe conditions as early as possible. Some studies have demonstrated that several blood markers could predict respiratory failure in COVID-19 patients [2,3]. Moreover, it has been reported that some inflammatory cytokines could distinguish disease severity in COVID-19 [4]. However, in some patients, the general condition dramatically worsens within a couple of days with severe respiratory failure. How to predict rapid respiratory failure remains unknown. Therefore, it is of high priority to identify reliable blood markers which could predict respiratory illness in the short term in clinical settings. This study aimed to investigate the predictive ability of laboratory markers for respiratory failure within the short term in COVID-19 patients.

## 2. Materials and Methods

### 2.1. Patients and Study Design 

This single-center, retrospective observational study was approved by the local ethics committee of Otsu City Hospital (No. 43). A total of 164 patients who tested positive for severe acute respiratory syndrome coronavirus 2 (SARS-CoV-2) by real-time polymerase chain reaction (PCR) from the nasopharyngeal swab were admitted to our hospital from 19 October 2020 to 30 April 2021. Data on demographic characteristics, underlying comorbidities, details of the treatment, time from symptom onset to admission, and the first measurement of blood specimens, and outcomes, were collected for all patients in this study. The laboratory values measured after admission included interleukin-6 (IL-6), C-reactive protein (CRP), lactate dehydrogenase (LDH), D-dimer, white blood cell (WBC), and creatinine (Cr) levels. Additionally, for 3 days from laboratory measurement, the fraction of inspired oxygen (FiO_2_) and the percutaneous oxygen saturation (SpO_2_) as a respiratory condition were assessed for all patients. For patients without invasive mechanical ventilation, the conversion of the inspired oxygen (O_2_) amount [liter (L)/minute (M)] to O_2_ concentration (FiO_2_ 0.25 to O_2_ 1 L/M, FiO_2_ 0.28 to O_2_ 2 L/M, FiO_2_ 0.32 to O_2_ 3 L/M, FiO_2_ 0.36 to O_2_ 4 L/M, FiO_2_ 0.4 to O_2_ 5 L/M) was used. Furthermore, the maximum FiO_2_ within 3 days was determined. The supplemental oxygen therapy was initiated when the patients’ SpO_2_ was less than 94%.

### 2.2. Laboratory Analysis of Blood Specimens

Serum and plasma samples were separated by centrifugation at 3000 rpm for 5 min. The levels of IL-6 were measured on a Cobas 8000 specimen form (Roche Diagnostics, Basel, Switzerland) by electrochemiluminescence immunoassay. LDH, Cr, and CRP levels were measured using a TBA-FX8 instrument (Canon Medical Systems, Tochigi, Japan) by an enzymatic reaction for LDH and Cr and latex turbidimetric reaction for CRP. WBC counts were performed using a DXH 900 hematology analyzer (Beckman Coulter, Tokyo, Japan). D-dimer levels were determined using a CS2100i automatic coagulation analyzer (Sysmex, Kobe, Japan) by a latex-enhanced photometric immunoassay.

### 2.3. Statistical Analysis

Categorical variables were presented as proportions or frequencies (%), and the χ^2^ test or Fisher’s exact test was used to compare the prevalence between the 2 groups. Normal and non-normally distributed continuous variables were presented as mean ± standard deviation and median (interquartile range) and were analyzed by variance analysis and Mann–Whitney U-test, respectively. Statistical significance was set at *p* < 0.05. The predictive value was evaluated by measuring the area under the curve (AUC). The optimal cutoff value was obtained by calculating the Youden index. Several cutoff values of each marker were obtained from different coordinates of the ROC curve to compare the sensitivity, specificity, positive predictive value (PPV), and negative predictive value (NPV) of these cutoff values. The odds ratio (OR) estimation was performed by using Fisher’s exact test. The correlation coefficients were obtained by the Spearman correlation analysis. Statistical analyses were performed using R software package (version 4.0.3, R Core Team. R: A language and environment for statistical computing. R Foundation for Statistical Computing, Vienna, Austria).

## 3. Results

### 3.1. Patient Characteristics

**Figure 1:**
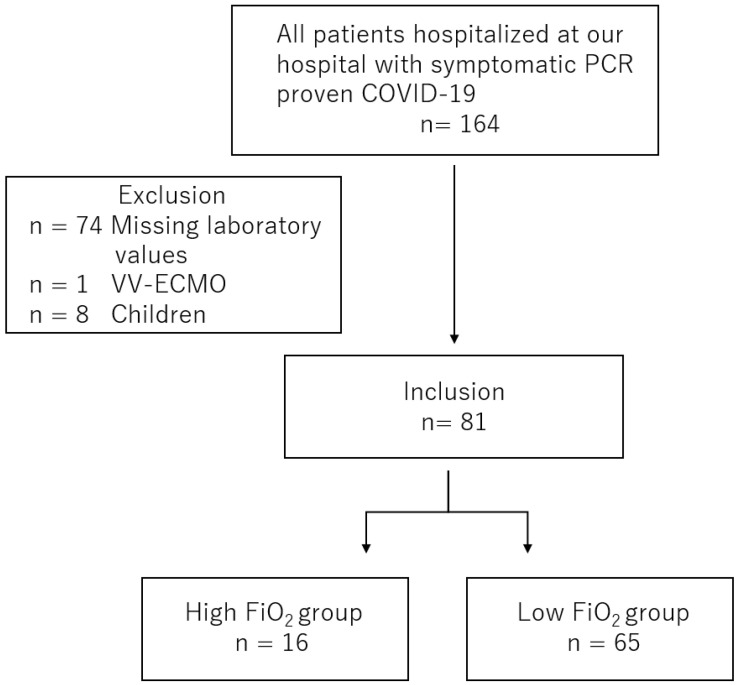
Study flow.

Among the 164 eligible patients, those lacking laboratory data (*n* = 74) and those younger than 18 years (*n* = 8) were excluded. One patient who received venovenous extracorporeal membrane oxygenation (ECMO) on the days of evaluation of laboratory values and oxygen amount was excluded. A total of 81 adult patients were included in the study (Figure 1). These patients were divided into a FiO_2_ ≥ 0.4 group (high FiO_2_ group (HFG) and a FiO_2_ < 0.4 group (low FiO_2_ group; LFG) according to the maximum FiO_2_ for three days from the measurement of laboratory markers. A total of 16 patients were assigned to the HFG group, and 65 patients were allocated to the LFG group (Figure 1). 

Among all patients, 50 patients (61.7%) were male, the mean age was 62.0 ± 16.3 years, and the mean body mass index (BMI) was 25.0 ± 4.5 kg/m^2^. There were no significant differences in sex, age, or BMI between the two groups. The number of comorbidities was significantly higher in the HFG patients. The most common comorbidity was hypertension (43.2%; Table 1). Most HFG patients (81.2%) received invasive mechanical ventilation, and all HFG patients were prescribed dexamethasone 6 mg daily (Table 1). Although many patients (65.4%) received favipiravir, any anti-inflammatory or anti-viral drug other than dexamethasone and favipiravir was not administered. The median times from symptom onset to admission and to the first measurement of each marker in the HFG patients were longer than those in the LFG patients. No significant difference in prognosis was found between the two groups (Table 1). In the HFG patients, the median time from evaluating the laboratory markers to exceeding the maximum FiO_2_ ≥ 0.4 was 1 (0–2) days. 

Because dexamethasone administration could be a significant confounding factor, a subgroup analysis focused on patients who received dexamethasone therapy. The 63 patients who received dexamethasone 6 mg daily were divided into 2 subgroups based on whether dexamethasone administration was initiated before or after the laboratory values were measured. A total of 28 patients received dexamethasone before the laboratory values were measured (preceding group). The remaining 35 patients were administered dexamethasone after the laboratory values were assessed (followed group). There were 10 and 6 patients assigned to the HFG in the preceding and followed groups, respectively. More patients in the HFG group received invasive mechanical ventilation in both the preceding and followed groups. No significant difference in the median time from symptom onset to dexamethasone therapy was found between the HFG and LFG groups (Table 2).

### 3.2. Laboratory Markers in COVID-19 Patients with Respiratory Failure 

First, among all patients, the levels of IL-6, CRP, LDH, WBC, D-dimer, and Cr in blood specimens of the HFG and LFG patients were compared. As shown in Table 1, the values of IL-6, CRP, LDH, WBC, D-dimer, and Cr were significantly higher in the HFG than in the LFG patients. To assess the diagnostic value of these parameters for respiratory failure with maximum FiO_2_ ≥ 0.4 within three days from laboratory measurement, ROC analysis was performed. The AUC value of IL-6, CRP, and LDH was high value of 0.85 [0.74–0.97], 0.82 [0.71–0.92], and 0.81 [0.70–0.92] with the cutoff value of 43.9, 5.7, and 268, respectively (Table 3). Among all patients, the OR value of each marker for the optimal cutoff value was evaluated. The crude OR of IL-6, CRP, and LDH showed the high value of 29.1 [5.6–295.6], 18.9 [3.8–188.4] and 9.5 [2.3–57.4], respectively (Table 4). That value of Cr showed no significance.

Second, among the patients who received preceding dexamethasone, the values of each laboratory marker for predicting respiratory failure were compared between HFG and LFG. IL-6 and CRP levels were significantly higher in the HFG than in the LFG (Table 2). Considering that the dexamethasone therapy may affect respiratory condition, the adjusted OR for each dexamethasone group was evaluated. The values of IL-6 and CRP adjusted for preceding dexamethasone were 53.9 [4.5–3242.8] and 16.1 [1.6–847.9], respectively (Table 4). The OR value of IL-6 and CRP adjusted for followed dexamethasone therapy showed the same value (Table 4). The adjusted OR value of WBC, D-dimer, and Cr showed no significance (Table 4).

Additionally, because our study aimed to evaluate the predictive ability of blood markers for respiratory deterioration within three days from analysis of these values, the cutoff point of IL-6, CRP, and LDH at which the value of PPV was the highest was determined. The cutoff value of IL-6 showed 91.5 with the corresponding PPV of 75.0% [42.8–94.5] (Table 5). The PPV value of CRP and LDH with the cutoff point of 6.7 and 305 was 50.0% [29.9–70.1] and 42.3% [23.4–63.1], respectively (Table 5).

**Figure 2:**
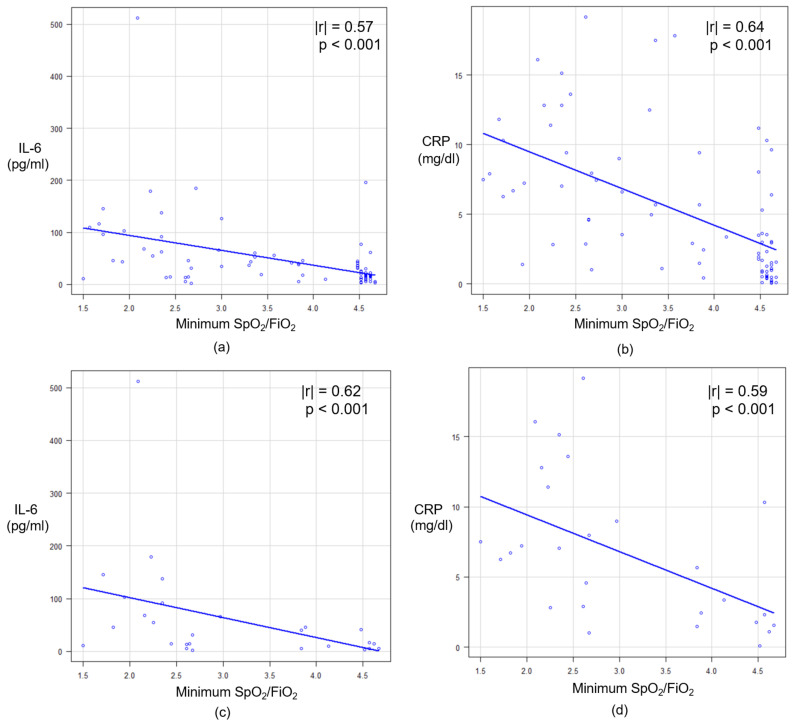
The correlation between minimum SpO_2_/FiO_2_ ratio and each blood marker: (**a**) IL-6 and (**b**) CRP among all patients; (**c**) IL-6 and (**d**) CRP in the preceding dexamethasone group. SpO_2_, percutaneous oxygen saturation; FiO_2_, fraction of inspired oxygen; IL-6, interleukin-6; CRP, C-reactive protein.

Furthermore, to reinforce the correlation between these blood markers and respiratory failure, the SpO_2_/FiO_2_ ratio was calculated as a respiratory functional parameter. As SpO_2_/FiO_2_ ratio would decrease by respiratory deterioration, the minimum value of SpO_2_/FiO_2_ ratio within three days from laboratory measurement was selected for the correlation analysis. Among the whole patients, the correlation coefficient value of CRP was higher than that of IL-6. For the preceding dexamethasone group, that value was higher in IL-6 than CRP (Figure 2).

## 4. Discussion

Similar to recent studies, our data showed that IL-6, CRP, LDH, WBC, and D-dimer levels were associated with the severity of COVID-19 infection within three days, which were significantly elevated in HFG compared with that in LFG. In the context of critical COVID-19 infection as multiple organ disease caused by cytokine response. Terpos et al. mentioned that high D-dimer levels might be associated with lethal disseminated intravascular coagulation (DIC)-related complications other than ARDS [5]. The WBC generally increases according to acute inflammation; this may reflect pulmonary and extrapulmonary organ damage, including respiratory distress, acute cardiac injury, and acute kidney injury. The high Cr levels directly indicate kidney injury. The severity of the disease might influence LDH levels because LDH is a general indicator of acute or chronic tissue damage that occurs in the heart, liver, lungs, muscles, and kidneys at high levels [2]. Our study revealed the high correlation coefficients of the serum level of IL-6 and CRP to minimum SpO_2_/FiO_2_ ratio, demonstrating that increase of the two markers indicates respiratory failure within three days after laboratory measurement. To find out the practical cutoff of these markers for respiratory failure, we set the outcome of this study on oxygen requirement with maximum FiO_2_ ≥ 0.4. The ROC analysis showed the high AUC and OR value of both IL-6 and CRP in the whole group. IL-6 level would be most useful with the highest value of AUC and the significant value of OR. In sub-group analysis, it was conspicuous that the median IL-6 level was high in the HFG patients of the preceding dexamethasone group. It can be explained by that this group might include a larger number of critical patients with respiratory collapse, causing earlier demand for dexamethasone therapy. The introduction of the dexamethasone therapy was decided by clinical physicians based on patients’ respiratory conditions and oxygen requirements. The dexamethasone therapy itself may not influence IL-6 level, which is supported by an analysis of the transcriptomic data that indicates the therapeutic mechanism of dexamethasone in severe COVID-19 patients does not involve IL-6 pathway [6]. The OR value of IL-6 adjusted for preceding dexamethasone administration showed a high value, indicating that IL-6 levels might reflect an ongoing respiratory deterioration in COVID-19 patients even under the dexamethasone therapy. On the contrary, previous reports have demonstrated that IL-6 receptor inhibitor therapy, such as tocilizumab, could prompt IL-6 synthesis to spike by receptor blockage [7]. Because our study excluded the cases with that therapy, the cutoff values of our result should not be applied to assess respiratory condition under IL-6 receptor inhibitor therapy.

Herold et al. demonstrated that elevated IL-6 levels predicted the need for mechanical ventilation [8]. IL-6 was found to be a precise marker for predicting respiratory illness, which is consistent with the highest OR in our study. As a result of this study, the optimal cutoff value of 43.9 for IL-6 showed the highest NPV of 96.4% [87.5–99.6]. Another possible cutoff value of IL-6 was 91.5, with the highest PPV of 75% [42.8–94.5]. These values of IL-6 might be used for classification of COVID-19 patients according to their prospective respiratory condition in the short term, for example, with a higher value of IL-6 than 91.5 for the respiratory deterioration group and a lower value of IL-6 than 43.9 for the stable group. However, it should be noted that the focus of our study was a respiratory condition within three days after assessing the laboratory data and that too early a decision on medical intervention by these markers may not be recommended. The evaluation of these biochemical markers requires appropriate subject and timing of the sample assessment and modification by physical parameters in the clinical context of COVID-19.

Our results suggest that IL-6, which is the key cytokine located upstream of the inflammatory cytokine cascade, increases prior to ARDS in critical COVID-19 patients, followed by an increase in acute-phase protein levels, such as CRP [9,10]. SARS-CoV-2 can rapidly activate pathogenic Th1 cells to secrete pro-inflammatory cytokines such as granulocyte-macrophage colony-stimulating factor (GM-CSF) and IL-6 [11]. The transition from viral pneumonia to ARDS is abrupt and recognizing potentially worsening patients among the stable majority must be a clinically urgent issue. Monitoring IL-6 as an early inflammatory marker for ARDS can be a useful tool for identifying collapsing patients in preparation for adequate interventions, particularly in pandemics with limited medical supplies.

A cytokine storm causing critical respiratory distress in COVID-19 is an immune disease characterized by high-level activation of immune cells and excessive production of massive inflammatory cytokines and chemical mediators [12]. Therefore, it has been suggested that the blockade of inflammatory cytokines in COVID-19 patients is a possible therapeutic tool. In clinical settings, many anti-inflammatory drugs, such as corticosteroids and IL-6 receptor inhibitors, are candidates for therapeutic strategies against COVID-19. However, the optimal timing for the administration of these drugs remains unclear. Too early administration can adversely lead to a decrease in viral clearance [13]. The use of corticosteroids has detrimental effects on the survival of patients not requiring oxygen [14]. IL-6 plays an important role in lung repair responses following viral insults, which means that the timing of administration of IL-6 receptor inhibitors could affect proper tissue remodeling [15]. Therefore, although the indication for medical interventions, including anti-inflammatory drugs administration, should not be based solely on IL-6 levels, this reliable marker could help physicians to judge appropriate timing for interventions. 

Recently, in Japan, out-of-hospital sudden death during home recuperation owing to COVID-19 pandemic is an emerging problem. The analysis for the COVID-19 patients dying at home or in a hotel for recuperation showed that the duration from COVID-19 diagnosis to death was 1–10 days (mean four days, median three days) [16]. Therefore, evaluating the IL-6 levels might contribute to the adequate decision on whether the patient should be admitted to hospital or not and subsequently to prevent out-of-hospital death.

This study had several limitations. First, this study was a single-center, retrospective observational study; thus, the number of cases was small, and other confounding factors might not have been considered. Second, since only six markers of blood specimens were measured, more sensitive markers that reflect inflammatory conditions may not be realized. Third, FiO_2_ was used as the parameter of the respiratory condition instead of the partial pressure of oxygen in arterial blood (PaO_2_). Although the PaO_2_/FiO_2_ (*p*/F) ratio might be more accurate for evaluating respiratory conditions, arterial blood gas analysis was not undertaken for all patients in this study. Finally, high-flow oxygen with FiO_2_ ≥ 0.4 was defined as respiratory failure, which might be the initial stage for acute respiratory distress.

## 5. Conclusions

The serum level of IL-6 is highly predictive of respiratory failure within three days in COVID-19 patients. Further studies are needed to investigate the validity of this inflammatory marker for predicting respiratory illness associated with COVID-19.

## Figures and Tables

**Table 1:** Clinical characteristics between the two groups.

Baseline Characteristics	Total (*n* = 81)	Maximum FiO_2_ ≥ 0.4		*p*-Value
		Yes (*n* = 16)	No (*n* = 65)	
Mean age, y (±SD)	62.0 ± 16.3	68.1 ± 13.9	60.6 ± 16.5	0.090
Male sex, *n* (%)	50 (61.7%)	12 (75.0%)	38 (58.5%)	0.260
Mean BMI, kg/m^2^ (±SD)	25.0 ± 4.5	25.7 ± 4.4	24.9 ± 4.5	0.500
Any comorbidities, *n* (%)	52 (64.2%)	15 (93.8%)	37 (56.9%)	0.007
Hypertension, *n* (%)	35 (43.2%)	10 (62.5%)	25 (38.5%)	0.100
Coronary artery disease, *n* (%)	8 (9.9%)	2 (12.5%)	6 (9.2%)	0.650
Diabetes mellitus, *n* (%)	18 (22.2%)	5 (31.2%)	13 (20.0%)	0.330
Chronic obstructive pulmonary disease, *n* (%)	7 (8.6%)	2 (12.5%)	5 (7.7%)	0.620
Bronchial asthma, *n* (%)	6 (7.4%)	0 (0%)	6 (9.2%)	0.590
Sleep apnea syndrome, *n* (%)	8 (9.9%)	2 (12.5%)	6 (9.2%)	0.650
Chronic kidney disease, *n* (%)	6 (7.4%)	3 (18.8%)	3 (4.6%)	0.088
Bacterial superinfection, *n* (%)	2 (2.5%)	1 (6.2%)	1 (1.5%)	0.360
Laboratory parameters				
Median IL-6 level, pg/mL (IQR)	24.5 (13.3–51.9)	93.8 (52.4–121.5)	18.0 (9.6–37.3)	<0.001
Median CRP level, mg/dL (IQR)	3.0 (1.1–7.9)	8.7 (7.0–12.1)	2.3 (0.9–5.7)	<0.001
Median LDH level, IU/L (IQR)	249 (203–329)	335 (285–484)	236 (193–300)	<0.001
Median WBC count, ×10^3^/mm^3^ (IQR)	5.6 (4.2–7.8)	8.3 (5.3–11.2)	5.1 (3.9–7.3)	0.003
Median D-dimer level, μg/mL (IQR)	0.7 (0.2–1.4)	0.9 (0.8–2.0)	0.5 (0.2–1.4)	0.048
Median Cr level, mg/dL (IQR)	0.8 (0.7–1.1)	1.1 (0.9–1.1)	0.8 (0.7–1.1)	0.027
Treatments				
Invasive mechanical ventilation, *n* (%)	14 (17.3%)	13 (81.2%)	1 (1.5%)	<0.001
Supplemental oxygen, *n* (%)	25 (30.9%)	3 (18.8%)	22 (33.8%)	0.370
Dexamethasone, *n* (%)	63 (77.8%)	16 (100%)	47 (72.3%)	0.017
Favipiravir, *n* (%)	53 (65.4%)	9 (56.2%)	44 (67.7%)	0.400
Median time from symptom onset to admission, days (IQR)	4 (2–7)	7 (5–7)	4 (2–6)	0.020
Median time from symptom onset to the first measurement of blood samples, days (IQR)	6 (4–9)	7 (6–10)	5 (3–9)	0.030
Prognosis				
Survivors, *n* (%)	76 (93.8%)	13 (81.2%)	63 (96.9%)	0.050
Non-survivors, *n* (%)	5 (6.2%)	3 (18.8%)	2 (3.1%)	

FiO_2_, fraction of inspiratory oxygen; BMI, body mass index; SD, standard deviation; IQR, interquartile range; IL-6, interleukin-6; CRP, C-reactive protein; LDH, lactate dehydrogenase; WBC, white blood cell; Cr, creatinine.

**Table 2:** Clinical characteristics between the two groups in the patients who received dexamethasone before or after measurement of blood markers.

	Preceding Dexamethasone Maximum FiO_2_ ≥ 0.4		*p*-Value	Following Dexamethasone Maximum FiO_2_ ≥ 0.4		*p*-Value
	Yes (*n* = 10)	No (*n* = 18)		Yes (*n* = 6)	No (*n* = 29)	
Median IL-6 level, pg/mL (IQR)	97.3 (58.1–144.0)	13.6 (4.9–27.3)	<0.001	79.2 (48.5–106.5)	31.7 (17.2–45.0)	0.054
Median CRP level, mg/dL (IQR)	7.4 (6.8–12.5)	2.7 (1.6–7.4)	0.018	9.9 (8.3–11.4)	3.0 (1.1–5.7)	0.023
Median LDH level, IU/L (IQR)	325 (263–455)	267 (210–349)	0.084	408 (301–585)	234 (199–270)	0.003
Median WBC count, ×10^3^/mm^3^ (IQR)	10.5 (6.1–12.6)	7.5 (6.4–9.9)	0.204	6.2 (4.7–8.3)	4.9 (4.0–6.3)	0.220
Median D-dimer level, μg/mL (IQR)	1.0 (0.8–2.9)	1.1 (0.4–1.8)	0.360	0.8 (0.4–1.0)	0.3 (0.1–1.2)	0.342
Median Cr level, mg/dL (IQR)	1.0 (0.8–1.1)	0.8 (0.8–1.1)	0.401	1.1 (1.1–1.2)	0.9 (0.7–1.1)	0.069
Invasive mechanical ventilation, *n* (%)	7 (70.0%)	0 (0.0%)	<0.001	6 (100%)	1 (3.4%)	<0.001
Supplemental oxygen, *n* (%)	3 (30.0%)	11 (61.1%)	0.236	0 (0.0%)	10 (34.5%)	0.152
Median time from onset to dexamethasone, days (IQR)	3.5 (2.0–5.0)	5.5 (3.0–10.0)	0.111	6.5 (6.0–8.5)	7.0 (5.0–8.0)	0.581

FiO_2_, fraction of inspiratory oxygen; IQR, interquartile range; IL-6, interleukin-6; CRP, C-reactive protein; LDH, lactate dehydrogenase; WBC, white blood cell; Cr, creatinine.

**Table 3:** Diagnostic characteristics of each marker to predict respiratory failure within three days from the measurement of these marker values.

Variable	AUC (CI)	*p*-Value	Cutoff	Sensitivity (%) (CI)	Specificity (%) (CI)	PPV (%) (CI)	NPV (%) (CI)
IL-6 level (pg/mL)	0.85 (0.74–0.97)	<0.001	43.9	87.5 (61.7–98.4)	81.5 (70.0–90.1)	53.8 (33.4–73.4)	96.4 (87.5–99.6)
CRP level (mg/dL)	0.82 (0.71–0.92)	<0.001	5.7	87.5 (61.7–98.4)	73.8 (61.5–84.0)	45.2 (27.3–64.0)	96.0 (86.3–99.5)
LDH level (IU/L)	0.81 (0.70–0.92)	<0.001	268	81.2 (54.4–96.0)	69.2 (56.6–80.1)	39.4 (22.9–57.9)	93.8 (82.8–98.7)
WBC count (×10^3^/mm^3^)	0.74 (0.61–0.87)	<0.001	7.8	56.2 (29.9–80.2)	81.5 (70.0–90.1)	42.9 (21.8–66.0)	88.3 (77.4–95.2)
D-dimer level (μg/mL)	0.66 (0.52–0.80)	0.023	0.7	81.2 (54.4–96.0)	58.5 (45.6–70.6)	32.5 (18.6–49.1)	92.7 (80.1–98.5)
Cr level (mg/dL)	0.68 (0.54–0.82)	0.012	0.9	66.7 (41.0–86.7)	58.5 (45.6–70.6)	30.8 (17.0–47.6)	86.4 (72.6–94.8)

IL-6, interleukin-6; CRP, C-reactive protein; LDH, lactate dehydrogenase; WBC, white blood cell; Cr, creatinine; AUC, area under the curve; CI, 95% confidence interval; PPV, positive predictive value; NPV, negative predictive value.

**Table 4:** The odds ratio of each marker for all patients and adjusted for preceding and followed dexamethasone therapy.

	Whole		Preceding Dexamethasone		Followed Dexamethasone	
Variable	Crude OR (CI)	*p*-Value	Adjusted OR (CI)	*p*-Value	Adjusted OR (CI)	*p*-Value
IL-6	29.1 (5.6–295.6)	<0.001	53.9 (4.5–3242.8)	<0.001	12.1 (1.1–646.6)	0.019
CRP	18.9 (3.8–188.4)	<0.001	16.1 (1.6–847.9)	0.006	12.1 (1.1–646.6)	0.019
LDH	9.5 (2.3–57.4)	<0.001	2.3 (0.4–18.0)	0.434	∞ (2.3–∞)	<0.001
WBC	5.5 (1.5–21.5)	0.004	2.8 (0.4–22.5)	0.254	4.1 (0.3–50.0)	0.195
D-dimer	6.0 (1.5–35.8)	0.005	5.4 (0.5–285.8)	0.194	3.6 (0.4–47.0)	0.191
Cr	2.8 (0.8–10.2)	0.068	4.4 (0.7–36.5)	0.114	3.4 (0.32–180.4)	0.377

IL-6, interleukin-6; CRP, C-reactive protein; LDH, lactate dehydrogenase; WBC, white blood cell; Cr, creatinine; OR, odds ratio; CI, 95% confidence interval.

**Table 5:** The cutoff value of three inflammatory markers with the highest positive predictive value.

Variable	Cutoff	Sensitivity (%) (CI)	Specificity (%) (CI)	PPV (%) (CI)	NPV (%) (CI)
IL-6 level (pg/mL)	91.5	56.2 (29.9–80.2)	95.4 (87.1–99.0)	75.0 (42.8–94.5)	89.9 (80.2–95.8)
CRP level (mg/dL)	6.7	81.2 (54.4–96.0)	80.0 (68.2–88.9)	50.0 (29.9–70.1)	94.5 (84.9–98.9)
LDH level (IU/L)	305	68.8 (41.3–89.0)	76.9 (64.8–86.5)	42.3 (23.4–63.1)	90.0 (80.0–97.0)

IL-6, interleukin-6; CI, 95% confidence interval; PPV, positive predictive value; NPV, negative predictive value.

## Data Availability

The data presented in this study are available on request from the corresponding author. The data are not publicly available because of privacy concerns.
